# Fibrocystin/polyductin (FPC): new functional insights into ARPKD pathogenesis revealed by informatics, comparative genomics, and model systems

**DOI:** 10.1007/s00467-025-07129-x

**Published:** 2026-01-28

**Authors:** Ashima Gulati, Ljubica Caldovic, Lisa M. Guay-Woodford

**Affiliations:** 1 Center for Precision Medicine and Genomics Research, Children’s National Research Institute, Children’s National Hospital, Washington, DC USA; 2 Department of Pediatrics, School of Medicine and Health Sciences, George Washington University, Washington, DC USA; 3 Children’s Hospital of Philadelphia, Roberts Center for Pediatric Research, 2716 South Street, Philadelphia, PA 19146 USA; 4 Department of Pediatrics, Perelman School of Medicine, University of Pennsylvania, Philadelphia, PA USA

**Keywords:** Autosomal recessive polycystic kidney disease (ARPKD), Fibrocystin/polyductin (FPC), Genotype-phenotype, Experimental models, Cilia-dependent cyst activation (CDCA) signal

## Abstract

Autosomal recessive polycystic kidney disease (ARPKD) is the prototype of the hepato-renal fibrocystic diseases, a subset of the broader ciliopathy disorders. As a severe form of PKD, ARPKD typically manifests in utero with 21% perinatal mortality and progressive loss of kidney function in most post-natal survivors. Congenital hepatic fibrosis is an invariant feature of ARPKD. *PKHD1*-encoded fibrocystin/polyductin (FPC) is a large 4074 amino acid glycoprotein that likely functions as a receptor molecule and appears to play a key role in maintaining differentiated renal tubular epithelium. The molecular mechanisms by which defects in FPC contribute to ARPKD pathogenesis are just beginning to be elucidated. FPC is a novel protein that likely evolved as vertebrates transitioned from aquatic to semi-terrestrial ecosystems. Full-length human FPC shares a phylogenetically conserved, multi-motif N-terminal region with its ancestral homolog, *PKHD1L1*, and contains a single-pass transmembrane domain and a novel C-terminal tail that harbors a ciliary targeting motif as well as mitochondrial and nuclear localization sequences. This review synthesizes the full range of recent experimental data about *PKHD1*/FPC to provide a current functional perspective about this complex protein. We also discuss the clinical relevance of these emerging functional insights for both kidney health and ARPKD pathogenesis.

**Graphical Abstract:**

**Figure:**
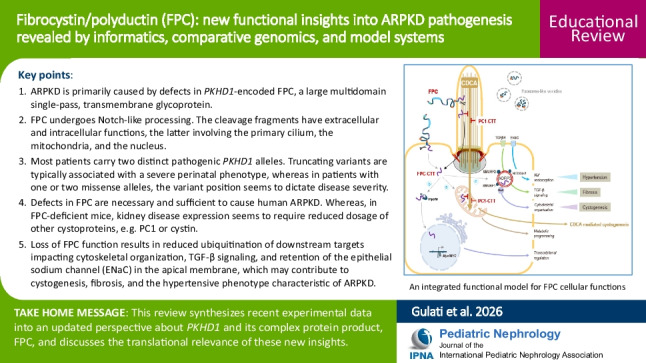
A higher resolution version of the Graphical abstract is available as Supplementary information.

**Supplementary Information:**

The online version contains supplementary material available at 10.1007/s00467-025-07129-x.

## Introduction

Autosomal recessive polycystic kidney disease (ARPKD; MIM 263200) is the most common form of the hepato-renal fibrocystic diseases, a set of monogenic ciliopathy disorders characterized by renal cystic disease and congenital hepatic fibrosis (CHF) or Caroli syndrome [1, 2]. Typically, ARPKD patients are identified either *in utero* or at birth due to enlarged, echogenic kidneys and oligo-/anhydramnios. The most severely affected neonates have a critical degree of pulmonary hypoplasia that is incompatible with survival [3, 4]. In a recent study, Alzarka et al. used electronic health record (EHR) data to calculate the annualized disease *incidence* as 1:26,485 live births and the perinatal mortality rate at 21% [5], suggesting that there are ~ 120 new ARPKD neonatal survivors per year in the United States. Based on *prevalence* calculations, the same study estimated that there are ~ 1500 patients (age 0–29 years) living with ARPKD in the US. The major post-natal morbidities include systemic hypertension, progressive kidney impairment, and portal hypertension, due to biliary dysgenesis (also known as ductal plate malformation (DPM)) [3, 6, 7], but the clinical course is highly variable [8, 9]. Of note, a small subset of patients with late-onset ARPKD have a liver-predominant phenotype and few or no manifestations of renal cystic disease [7].

Essentially all cases of ARPKD involve mutations in *PKHD1*, which encodes fibrocystin/polyductin (FPC), a very large, single-pass, transmembrane glycoprotein [10, 11]. There do not appear to be mutational hotspots, and most patients carry two disease-causing alleles (compound heterozygotes). In addition, a small subset of patients (< 1%) develop an ARPKD-like phenotype due to pathogenic variants in either *DZIP1L* (encoding DAZ interacting protein 1-like protein) [12] or in *CYS1* (encoding Cystin-1)*, *the human orthologue of the gene disrupted in the *Cys1*^*cpk/cpk*^* (cpk*) mouse model, in which the renal cystic disease closely phenocopies ARPKD [13].

While *PKHD1* was identified as the principal ARPKD gene over 20 years ago, relatively little is known about the molecular functions of its gene product. This review synthesizes the current information about FPC, its putative phylogenetic origin, predicted molecular structure and post-translational processing, cellular expression, and proposed functional domains and molecular interactions, providing new insights about how this complex protein functions in kidney health and ARPKD pathogenesis.

## FPC: phylogenic insights

Clues about protein function are often suggested by the ontogeny of a protein family and assessment of phylogenetic and sequence conservation among its members. The FPC family includes the phylogenetically novel proteins, FPC and FPC-L, the latter encoded by *PKHD1L1* [14]. FPC-L is a component of the stereocilia coat in cochlear hair cells, which are essential for hearing. Defects in *PKHD1L1* cause autosomal recessive, non-syndromic, mild-moderate to severe sensorineural hearing loss [15].

Based on informatic analysis, Harafuji et al. [16] showed that human FPC-L has 25.0% identity and 41.5% similarity to the human FPC extracellular N-terminal domain (NTD). Protein motifs within the extracellular domain appear to be relatively conserved, with putative orthologues identified across eukaryotes from single-celled organisms, such as green algae, to vertebrates [17]. However, there is no significant sequence similarity between the FPC-L and FPC transmembrane (TM) domains and these two proteins have markedly different cytoplasmic tails (CTT)—9 amino acid (AA) in FPC-L versus 192 AA in FPC, with the latter being unique among vertebrate proteins [16]. Taken together, these data suggest *PKHD1L1*-encoded FPC-L is the ancestral member of this novel protein family.

Query of the NCBI nr database with human FPC has revealed putative orthologs in 66 mammalian, 27 bird, 5 reptile, 3 amphibian, and 1 fish species. Alignment of these 102 FPC sequences demonstrated higher conservation across the extracellular FPC-NTD than the cytoplasmic FPC-CTT [16]. Specifically, pairwise comparison of human and mouse FPC revealed 73% overall sequence identity, but only 55% identity across the FPC-CTT [18]. This limited conservation of the FPC-CTT suggests that intracellular FPC functions may differ among species that occupy different ecological niches.

## FPC: protein structure and post-translational processing

### Protein structure

**Fig. 1:**
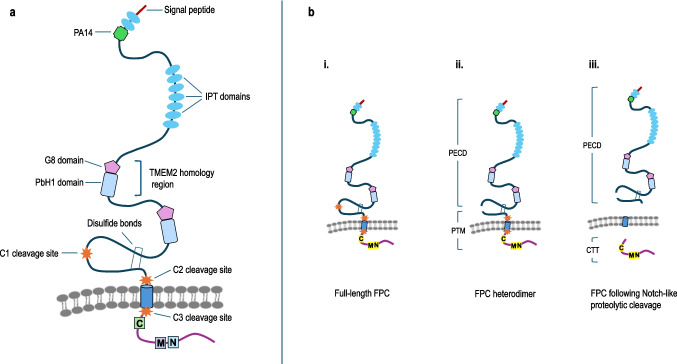
FPC protein structure and post-translational processing: **a** Schematic showing the FPC domain architecture (see text and Table 1 for domain descriptions); **b** FPC post-translational processing (i) full-length FPC, (ii) FPC is cleaved at the proprotein convertase (C1) site producing an extracellular fragment (polyductin extracellular domain, PECD) and a fragment that includes the transmembrane domain with the intracellular domain (PTM), (iii) juxtamembrane cleavage of the PTM by ADAM metalloproteases (C2 site) sheds the PECD into the urine. Further intramembranous cleavage (C3) by γ-secretases releases the intracellular C-terminal tail (CTT). Modified from [17, 22]

Human FPC is a type I membrane protein of 4074 AAs comprising a large, highly glycosylated NTD (AA 19–3858), a single-pass TM domain (AA 3859-3881), and a 192 AA cytoplasmic CTT [17] (Fig. 1a).

While the crystal structure of FPC has not been solved, informatic analyses indicate that FPC is a novel protein with a unique combination of conserved domains found among proteins that function as receptor molecules [10, 17, 19] (Table 1). Specifically, the FPC-NTD is predicted to contain a protective antigen 14 (PA14) domain [20], 12 immunoglobulin-like plexin-transcription factor (IPT)/IPT-like domains, a sperm protein, enterokinase and agrin (SEA) domain, and two regions of transmembrane protein 2 (TMEM2) homology. The PA14 domain has been implicated in binding to glycoproteins, while the IPT domains may facilitate ligand-binding, modulate FPC-NTD oligomerization, and/or mediate protein–protein interactions [17]. TMEM2 functions as a cell surface hyaluronidase [21]. The regions of TMEM2 homology in FPC consist of two novel G8 domains (each with 8 conserved glycine residues) immediately followed by multiple parallel beta-helix 1 (PbH1) repeats [10, 11]. This tandem configuration suggests the FPC-NTD may have hyaluronidase-like activity as well as a carbohydrate-binding function [17, 21].

**Table 1:** FPC predicted domains

AA position	Structural motif	Comment
258–355	IPT-domains	IPT-PA14 region
322–485	PA14	
945–1001	IPT domain	Extensive IPT domains may facilitate peptide binding
1018–1103	IPT domain	
1107–1193	IPT domain	
1195–1294	IPT domain	
1388–1482	IPT domain	
1486–1565	IPT domain	
1572–1659	IPT domain	
1932–2053	G8 domain	G8-PbH1 region may facilitate carbohydrate binding
2226–2248	PbH1 domain	
2249–2271	PbH1 domain	
2292–2325	PbH1 domain	
2326–2347	PbH1 domain	
2409–2431	PbH1 domain	
2469–2502	PbH1 domain	
2740–2873	G8 domain	Second G8-PbH1 region may represent a tandem duplication
3010–3032	PbH1 domain	
3033–3055	PbH1 domain	
3086–3108	PbH1 domain	
3557–3717	SEA domain	Contains C2 cleavage site
3859–3881	TM region	Single pass hydrophobic helix
3882–4074	Low complexity	C-terminal tail contains a ciliary targeting sequence (CTS), as well as mitochondrial and nuclear localization sequences (MLS and NLS, respectively). Mitochondrial (MLS) and nuclear (NLS) localization sequences

PA14: protective antigen 14; IPT: immunoglobulin-like plexin-transcription factor; PbH1: parallel beta-helix 1; SEA: sperm protein, enterokinase and agrin; TM: transmembrane. Modified from Bannell [17]

### Post-translational processing

FPC is proteolytically cleaved through a regulated and Notch-like processing mechanism externally and internally [17, 22] (Fig. 1). Experimental data from several studies suggest that FPC is synthesized as a single polypeptide. During its processing through the secretory pathway, about 50% of the full-length protein remains intact and the remainder is cleaved by a putative proprotein convertase (Fig. 1a: C1 cleavage site) [22], resulting in an extracellular fragment (polyductin extra-cellular domain, PECD) and transmembrane with the CTT fragment (PTM) that are linked as a heterodimer by disulfide bonds at the cell surface (Fig. 1b). Both the full-length FPC and the heterodimer are trafficked to the ciliary and apical membranes. Binding of an as yet to be defined ligand(s) triggers juxtamembrane proteolytic cleavage by ADAM proteases within the SEA domain (Fig. 1a: C2 cleavage site), releasing the PECD to be shed into the urine as either a soluble protein or incorporated into urinary exosome-like vesicles (ELV) with two other major cystoproteins, polycystin–1 (PC1) and polycystin-2 (PC2), forming the polycystin protein complex [23, 24]. The former may act downstream in the renal tubule as a soluble urinary signaling molecule, whereas the exosome-bound polycystin protein complex may mediate urocrine signaling by inserting into the ciliary membrane of more distal tubular epithelia [17].

Intramembrane cleavage within the TM domain by γ-secretase (Fig. 1a: C3 cleavage site) releases the FPC-CTT, which contains a ciliary targeting sequence (CTS) [25], as well as mitochondrial and nuclear localization sequences (MLS and NLS, respectively) [26, 27] and at least three PKA/PKG phosphorylation sites [10, 11]. Following enzymatic cleavage, the FPC-CTT translocates to the nuclear and/or mitochondrial compartments.

#### Translational relevance

Notch-like processing suggests that FPC has distinct extracellular and intracellular signaling functions. The PECD, containing most of the extracellular NTD, may have paracrine signaling functions, either as a soluble fragment or as part of the ELV-bound polycystin complex. The FPC-CTT targets to multiple intracellular compartments and contributes to ciliary signaling, mitochondrial function, and transcriptional regulation. Defining each of these FPC-related processes will help identify novel druggable targets for therapeutic intervention.

## FPC: tissue expression and subcellular localization

During fetal development, *PKHD1* is broadly expressed in multiple tissues including the mesonephric tubules, ureteric bud, immature hepatocytes, primordial gut, adrenal cortex, neural tube, developing heart, and bronchi, suggesting that FPC plays a role in organ development and tubular morphogenesis [11, 18] and consistent with the hypothesis that ARPKD pathogenesis involves a defect in epithelial terminal differentiation [9]. In adult tissues, immunohistochemical analyses have demonstrated that FPC is most prominently expressed in renal collecting ducts as well as ductal structures in the liver and pancreas [23, 24, 28, 29], suggesting an as yet undefined role for FPC in maintaining the integrity of terminally differentiated ductal epithelia [28, 30].

RNA analyses indicate that mouse *Pkhd1* is transcriptionally complex, more so in the kidney than in the liver [31, 32]. In mouse renal epithelia, extensive alternative splicing generates multiple polysome-associated mRNA transcripts, suggesting that these cells have multiple protein isoforms [31]. However, similar transcriptional complexity has not been demonstrated for human *PKHD1*, in either the kidneys or the liver.

At the subcellular level, FPC localizes to the primary apical cilia and basal bodies in renal tubular and biliary ductal epithelia, as well as to their apical membranes and nuclei [23, 26, 33–36]. In proliferating cells *in vitro,* FPC-CTT localizes to the centrosomes and mitotic spindles [37].

## FPC: insights from genetics

### Human ARPKD: *PKHD1* genotype-phenotype correlations

In selected ARPKD cohorts, underlying pathogenic *PKHD1* variants have been detected in 81–87% of probands analyzed [9]. Overall, more than 1400 pathogenic and likely pathogenic sequence variants have been identified along the entire length of the *PKHD1* gene and catalogued in ClinVar (www.ncbi.nlm.nih.gov/clinvar/). While there is no mutational hot spot *per se*, a missense mutation in exon 3, c.107C > T (p.Thr36Met), accounts for approximately 15–20% of all mutant alleles [38].

Most patients with ARPKD are compound heterozygotes for *PKHD1* sequence variants; that is, they carry two different disease-causing alleles. Those with two truncating variants typically have a severe phenotype leading to perinatal or neonatal mortality, whereas missense mutations, resulting in an AA change, are typically associated with neonatal survival [9, 38, 39]. No substantial clinical differences have been described between patients with two missense mutations and those with missense and truncating mutations in *trans*, suggesting that the missense mutations may dictate the disease severity since those are likely to produce a full-length protein with residual function [40, 41].

**Fig. 2:**
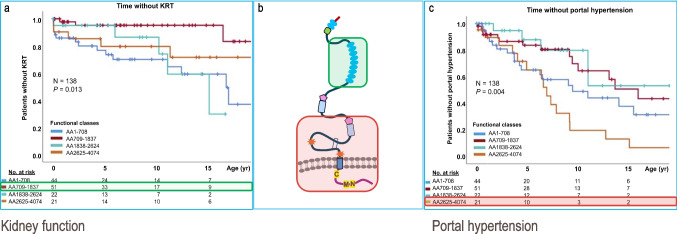
Correlation of FPC variant position with outcomes: **a** Kaplan–Meier survival without kidney replacement therapy; **b** FPC schematic representation with outcome correlations between variant position and kidney function (green box) or portal hypertension (red box); **c** Kaplan–Meier survival without portal hypertension Modified from Burgmaier et al. [41]

In a recent study of over 240 patients with bi-allelic *PKHD1* variants, the European ARegPKD consortium reported that the *position* rather than *type* of *PKHD1* variant may correlate with the ARPKD clinical phenotype. Patients with two missense variants affecting FPC AA 709–1837, corresponding to the IPT domains (Fig. 2) or a missense variant in this region combined with a null variant less frequently developed early kidney failure. With respect to liver disease, patients with missense variants affecting FPC AA 1838-2624 presented with less severe liver disease, whereas those with variants affecting AA 2625-4074, which includes the second G8/PbH1 domain, TM region, and CTT (Fig. 2) had more severe liver disease, as measured by signs of portal hypertension (e.g., thrombocytopenia, splenomegaly, or varices) or substantial hepatic complications (e.g., liver transplantation, variceal bleeding, interventional or surgical generation of portosystemic shunts) [41].

Such genotype–phenotype correlations may inform the generation of new hypotheses about FPC function and the significance of the different protein domains. However, notable exceptions to these patterns emphasize the complexity of FPC pathobiology. For example, children homozygous for *PKHD1* deletions or truncating mutations are known to survive well past the neonatal period [42, 43]. Moreover, not all missense mutations lead to a milder disease [38], suggesting that missense variants, perhaps due to their position within protein structure, can have a diverse impact on protein function. Genetic and environmental modifiers likely also play a significant role in disease expression, as illustrated by significant phenotypic variability in subsets of sibships [6].

#### Translational relevance

Most patients with ARPKD carry two distinct pathogenic *PKHD1* alleles. While the missense mutation in exon 3, c.107C > T (p.Thr36Met), accounts for approximately 15–20% of all *PKHD1* mutant alleles, there are no mutational hot spots per se in *PKHD1*, unlike *CFTR* where the F508-del mutation accounts for > 70% of all mutant alleles [44]. As might be expected, patients with two truncating *PKHD1* variants typically have a severe phenotype leading to perinatal or neonatal mortality. In contrast, in patients with one or two missense variants, the kidney and liver disease severity appears to be dictated by the *position* of the *PKHD1* variant. If confirmed by more refined analyses in larger ARPKD cohorts, this genotype-phenotype information may more precisely inform prognostic counseling for affected patients.

#### Monoallelic (heterozygous) *PKHD1* variants

Monoallelic (heterozygous) *PKHD1* variants are not sufficient to cause classic ARPKD. However, subsets of parents (obligate heterozygotes) with children who have *PKHD1*-related ARPKD may express mild or subclinical manifestations, including small renal cysts, increased cortical echogenicity, or hepatic cystic changes [45]. In other cohorts, monoallelic *PKHD1* variants have been associated with dominant-appearing polycystic liver disease or atypical cystic kidney phenotypes, suggesting partial penetrance or a dosage-sensitive effect. Moreover, *PKHD1* heterozygosity may act as a genetic modifier, interacting with disease-causing variants in other cystic disease genes such as *PKD1*, thereby influencing disease expressivity and severity [46].

These findings suggest that while monoallelic *PKHD1* variants do not cause classic ARPKD, they may contribute to milder or atypical hepatorenal cystic phenotypes and may serve as genetic modifiers within the broader cystic disease spectrum. Validation of these hypotheses will require further systematic assessments in larger cohorts.

#### Experimental rodent models

Experimental rodent models often provide a critical resource to elucidate the function of gene products and pathogenic pathways. However, orthologous *Pkhd1* rodent models do not faithfully phenocopy the human cystic kidney disease, thus constraining the functional evaluation of FPC and suggesting that the protein function may differ from species to species.

In the rat, a spontaneously occurring variant (IVS35-2A → T) arose in the Crj:CD/SD strain, causing skipping of *Pkhd1* exon 36 with a consequent frameshift and premature termination of the protein. When homozygous, this *Pkhd1*^*pck*^ (*pck)* allele causes biliary dysgenesis/the DPM lesion *in utero*. However, the kidneys are normal at birth. Renal tubular dilatation begins 2–3 weeks postnatally, gradually involving the thick ascending loops of Henle (TAL), distal tubules, and cortical collecting ducts (CD), and resulting in slowly progressive PKD [10, 47]. Indeed, in the initial characterization of its disease phenotype, the *pck* rat was proposed as a model of autosomal dominant PKD (ADPKD) [47]. At the cellular level, the loss of FPC causes malformed cilia in the intrahepatic bile duct cholangiocytes [36], providing further evidence that ARPKD is a hepato-renal ciliopathy.

Multiple mouse *Pkhd1* models have been reported. Most were genetically engineered [48] and one arose spontaneously [32] (Table 2). While all these models express DPM-related liver disease, an early-onset renal cystic phenotype is either lacking and/or strain-dependent, suggesting that the mouse has reno-protective mechanisms that are not expressed in humans with *PKHD1* variants. Given the transcriptional complexity of mouse *Pkhd1* in renal epithelia [31], a logical consideration is that alternative *Pkhd1* isoforms may partially compensate for the loss of full-length FPC, whereas in the liver, where *Pkhd1* is less transcriptionally complex [32], the alternative isoforms expressed in cholangiocytes may not be functionally sufficient.

**Table 2:** Discordant species-specific renal phenotypes with *Pkhd1/PKHD1* defects

	**Strain**	**Kidney**	**Liver**	**Pancreas**	**Ref**
**Mouse *Pkhd1* mutants**					
* Pkhd1*^lacZ/lacZ*^	129 Sv/C57BL6J	PT dilatation	DPM	Cystic	[75]
* Pkhd1*^*del2/del2*^	BALBc/C57BL/6 J	PT dilatation	DPM	Cystic	[76]
* Pkhd1*^lsl/lsl**^	129 Sv/C57BL6J	TAL/CD dilatation	DPM	Cystic	[23]
* Pkhd1*^*del3−4/del3−4*^	129 Sv/C57BL6J	TAL/CD dilatation	DPM	Cystic	[50]
* Pkhd1*^*del3−67/del3−67*^	C57BL6J	None	DPM	Cystic	[49]
* Pkhd1*^*del4/del4*^	129 Sv/C57BL6J	None	DPM	Cystic	[77]
* Pkhd1*^*C642/*+^	C57BL/6 J	PT dilatation^a^	DPM	-	[78]
* Pkhd1*^*de15−16GFP/de15−16GFP*^	C57BL/6 J	Tubular dilatation	DPM	Cystic	[79]
* Pkhd1*^*cyli/cyli*^	DBA/2 J	None	DPM	None	[32]
* Pkhd1*^*del67/del67*^	C57BL/6 J	None	None	None	[24]
**Rat *Pkhd1* mutant**					
* Pkhd1*^*pck/pck*^	Crj:CD/SD	TAL/CD dilatation	DPM	None	[47]
**Human *PKHD1* patients**					
		TAL/CD dilatation	DPM	None	[9]
		None^b^	DPM	None	[7]

*Replacement of exons 1–3 with LacZ reporter; **Insertion of lox-stop-lox cassette in IVS2; PT: proximal tubule; TAL: thick ascending limb; *CD*, collecting duct; *DPM*, ductal plate malformation (portobiliary dygenesis); ^a^PT cysts develop in aged (aged > 15mo) homozygotes as well as heterozygote *females;* ^b^Very small number of patients

Observations from two recently generated mouse models add further complexity to the *PKHD1/Pkhd1* genetic enigma. The *Pkhd1*^*del67/del67*^ model, which essentially lacks the CTT, does not express a renal, biliary, or pancreatic phenotype, even in mice aged more than 18 months [24]. In comparison, the *Pkhd1*^*del3−67/del3−67*^ model, which lacks most of the FPC protein coding region, has no renal histopathological phenotype but does express the DPM lesion and dilated pancreatic ducts [49], indicating that *Pkhd1* plays an important but complicated role in the differentiation and maintenance of ductal epithelia.

Taken together, these observations suggest that the limited/absent renal phenotype in the mouse cannot be directly attributed to intragenic factors, such as the generation of alternative isoforms, and that expression of the renal cystic phenotype may involve more complex mechanisms, e.g., genetic or environmental modulators. In support of this thesis, comparison of *Pkhd1*^*del3−4/del3−4*^ mice vs. *Pkhd1*^*del3−4/del3−4*^; *Pkd1*^+*/−*^ mice revealed that the digenic mutants express more severe renal cystic disease, suggesting that *Pkd1* gene dosage modulates ARPKD pathogenesis in the mouse [50]. Studies by Olson et al. [51] further demonstrated that combining a knockout (KO) of the *Pkhd1* gene (*Pkhd1*^*lsl/lsl*^) with a hypomorphic *Pkd1* allele in both mice and rats resulted in a severe, rapidly progressive renal cystic disease like human ARPKD.

Studies by Zhang et al. [52] have further demonstrated the modulating role of other cystoproteins on FPC function. In kidneys and isolated renal epithelial cells from *cpk* mice, they showed that the absence of cystin is associated with a significant reduction in FPC, a functional interaction that is unidirectional since cystin abundance is not altered in mice lacking functional FPC. This study provides potentially new insights into human ARPKD pathogenesis, given that a handful of patients with an ARPKD-like phenotype have been reported with bi-allelic defects in *CYS1* [13]. In addition, Mrug et al. [53] identified the ciliary protein, Kif12, as a putative genetic modifier of renal cystic disease in the *cpk* mouse, thus expanding the evidence base for genetic modulation in ARPKD pathogenesis.

##### Translational relevance

The lack of a kidney phenotype in *Pkhd1* mutants may suggest that mice, unlike humans, have a renoprotective mechanism, the elucidation of which could provide new insights for therapeutic targeting. In addition, haploinsufficiency of *Pkd1* in rodent *Pkhd1* mutants unmasks a kidney phenotype, which may suggest that the respective proteins, FPC and PC1, function, at least in part, in common pathways. This observation provides a rational basis for evaluating therapies developed for ADPKD as potential therapeutic agents in ARPKD.

#### Preclinical trials in the *pck* rat

A large body of evidence has demonstrated that arginine vasopressin (AVP) and AVP V2 receptor signaling promote cystic kidney disease progression in human ADPKD as well as rodent PKD models, and V2 receptor antagonists inhibit cystogenesis by downregulating cAMP signaling and cell proliferation [54]. Two studies have examined the relevance of cAMP signaling in cystic kidney disease progression in the *pck* rat, an orthologous model of human ARPKD. In the first study, administration of OPC-41061 (tolvaptan), a V2 receptor antagonist used in humans as a treatment for hyponatremia and congestive heart failure, significantly attenuated cystic kidney disease progression in treated vs. untreated *pck* rats [55]. In the second, genetic elimination of circulating vasopressin significantly attenuated the development of cystic kidney disease in *pck* rats, and this effect was reversed by administration of the V2 receptor agonist, 1-deamino-8-d-arginine vasopressin (DDAVP) [56]*.* While the specific mechanism(s) by which the loss of functional FPC triggers a V2 receptor-mediated increase in cAMP signaling remains to be elucidated, the combination of these pharmacological and genetic data has provided the rationale for current clinical trials evaluating tolvaptan in patients with ARPKD [57].

## FPC: functional insights

While the function of FPC remains incompletely understood, experimental data suggest that this glycoprotein plays key roles in several cellular processes, including (i) ciliary sensing and signaling; (ii) transcriptional regulation and nuclear signaling pathways; (iii) ubiquitination; (iv) mitochondrial function; (v) cellular adhesion; and (vi) regulation of inflammation and fibrosis. These functional roles are described in brief below.

### Ciliary sensing and signaling

FPC plays an important role in establishing and maintaining normal ciliary architecture [36, 52]. From a functional perspective, FPC appears to function as a ciliary sensor with the binding of a yet-to-be-defined ligand(s) triggering its Notch-like proteolytic cleavage. FPC and the polycystins, PC1 and PC2, co-localize in the primary apical cilium [34, 58]. Initial studies indicated that the FPC-CTT interacted with PC2, suggesting a possible common cystogenic pathway for both ARPKD and ADPKD [59, 60]. However, this putative interaction has been called into question by the failure to co-immunoprecipitate PC2 from mouse kidneys expressing FPC with a C-terminal epitope tag [24].

**Fig. 3:**
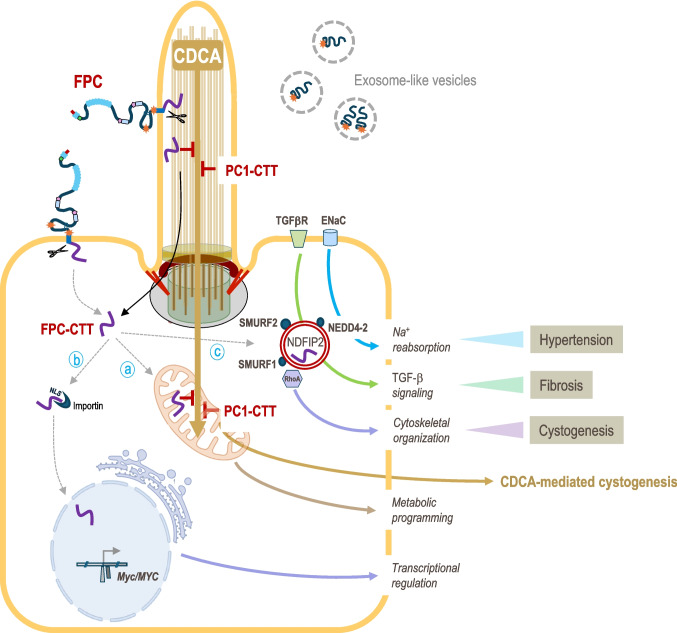
An integrated functional model for FPC cellular functions. FPC localizes to the primary apical cilium and undergoes Notch-like processing that releases its extracellular PECD and intracellular CTT. The FPC-PECD is shed into the urine as either a soluble protein or incorporated into urinary exosome-like vesicles (ELV) with two other major cystoproteins, polycystin-1 (PC1), and polycystin-2 (PC2), forming the polycystin protein complex. Both the soluble PECD and the PECD-containing ELV may function in urine-facing, extracellular signaling pathways. The FPC-CTT has at least 3 proposed functions: **a** it acts synergistically with the PC1-CTT to repress the ciliary component of the cilia-dependent cyst activation (CDCA) signal and inhibit the propagation of the CDCA signal in the mitochondria. In addition, the FPC-CTT plays a key role in maintaining the integrity of mitochondrial structure and metabolic function; **b** The FPC-CTT complexes with Importinβ1 and translocates to the nucleus where it may play a role in transcriptional regulation, including *Myc* expression; and **c** The FPC-CTT may play a role in regulating ubiquitination pathways. The FPC-CTT co-localizes in intracellular vesicles with NDFIP2, a key adaptor and regulator of the HECT family of E3 ubiquitin ligases. Loss of FPC function alters the subcellular localization of the E3 ubiquitin ligases, SMURF1, SMURF2, and NEDD4, resulting in reduced ubiquitination of downstream targets causing increased TGF-β signaling that could contribute to fibrosis, enhanced epithelial sodium channel (ENaC) retention in the apical membrane, leading to increased Na^+^ reabsorption that could contribute to systemic hypertension, increased RhoA expression and disruption of cytoskeletal organization that could contribute to cystogenesis. Modified from [16, 27, 66] with figure components created using BioRender

While there is no current evidence of physical interaction between FPC and the polycystins in mouse kidneys [51], Walker et al. [27] have proposed a new ciliary interaction in which FPC and PC1 cooperatively function to repress the cilia-dependent cyst activation (CDCA) signal (Fig. 3).

### Transcriptional regulation and nuclear signaling pathways

The FPC-CTT contains a functional nuclear localization sequence [26]. Data from complementary studies using immunolocalization [26], tagged FPC constructs [23, 24], and single-particle electron microscopy [61] have demonstrated that the FPC-CTT localizes to the nucleus and binds to DNA (Fig. 3).

However, the role that FPC plays in transcriptional regulation presents a paradox. While *in vitro* studies have demonstrated that both human and mouse FPC-CTT can localize to the nucleus, bind, and activate the *MYC/Myc* P1 promoter, *in vivo *comparative analyses revealed that *MYC* expression is upregulated in kidneys from ARPKD patients but not altered in kidneys from several *Pkhd1* mutant mice [16]. This paradox may be explained in part by the temporal, species-specific differences in the expression of *PKHD1/Pkhd1 and CYS1/Cys1* in developing mouse vs. human kidneys. *Cys1* expression is activated before *Pkhd1* in developing mouse kidneys, whereas the reverse sequence of expression is observed in developing human kidneys [16]. Given that cystin, the *Cys1* gene product, is a negative regulator of *Myc* transcription, this temporal difference in expression may help explain normal *Myc* expression in *Pkhd1* mutant kidneys and elevated *MYC* expression in kidneys from ARPKD patients [62].

In human ARPKD kidneys, the transcription factor STAT3 is upregulated in cyst-lining renal epithelial cells [63]. In cultured HEK293T cells, the FPC-CTT translocates to the nucleus and complexes with SRC, inhibiting its activation as well as SRC-activated STAT3 signaling, suggesting that aberrant STAT3 signaling is an important contributor to *PKHD1*-related cystogenesis [63]. In addition, human FPC may be subject to autoregulation, as studies in HEK293 cells have demonstrated that the human FPC-CTT antagonizes the regulation of the PI3K/Akt/mTOR pathway mediated by full-length FPC and promotes cystogenesis *in vitro* [64].

Taken together, these studies suggest that the FPC-CTT modulates nuclear signaling pathways that are dysregulated in ARPKD, perhaps in a species-specific fashion.

### Ubiquitination

Ubiquitination is a cellular signaling mechanism that regulates the function and levels of multiple proteins [65]. Kaimori et al. [66] were the first to show that FPC plays a role in regulating ubiquitination pathways. Specifically, they demonstrated that the FPC-CTT co-localizes in intracellular vesicles with NDFIP2, a key adaptor and regulator of the HECT family of E3 ubiquitin ligases (Fig. 3). Loss of FPC function altered the subcellular localization of at least three different HECT-E3 family members: SMURF1, SMURF2, and NEDD4, resulting in reduced ubiquitination of downstream targets causing: (i) increased RhoA expression and disruption of cytoskeletal organization that may contribute to cystogenesis; (ii) increased TGF-β signaling that may contribute to kidney/portal tract fibrosis; and (iii) enhanced epithelial sodium channel (ENaC) retention in the apical membrane of renal CD cells, leading to increased Na reabsorption that may contribute to severe systemic hypertension. While upregulation of RhoA, TGF-β signaling, and ENaC activity has been reported in ARPKD renal and biliary epithelial cells, the mechanism through which FPC modulates NDFIP2 localization and disrupts its function remains to be defined.

A set of complementary studies further support a role for FPC in regulating ubiquitination. While *Pkhd1*^*del3−67/del3−67*^ mice lacking essentially the entire FPC coding sequence do not exhibit renal cysts, single nucleus RNA-seq analysis of their kidneys revealed dysregulation of the ubiquitination pathway [67]. In addition, Zhang et al. [52] described selective defects in ubiquitination and protein homeostasis in the kidneys, as well as cortical CD cells isolated from *cpk* mice, likely due to post-translational loss of FPC. This latter study demonstrated that cystin is required for maintaining FPC levels, a functional interaction that appears to be important in safeguarding proteome integrity in mouse renal epithelial cells.

### Mitochondrial function

As in ADPKD, bioenergetic remodeling has been implicated in ARPKD pathogenesis [68]. Genetically engineered HEK293 cells carrying clinically identified *PKHD1* truncating variants displayed mitochondrial structural abnormalities and increased rates of cellular oxygen consumption and extracellular acidification, suggesting roles for FPC in mitochondrial structural integrity and energy metabolism regulation [69]. These findings recently have been extended by Walker et al. [27] who demonstrated (i) the intracellular C-terminal fragment 15 (ICD_15_) of both human and mouse FPC-CTT contains a functional mitochondrial localization sequence (MLS); (ii) in both *Pkhd1* kidneys lacking FPC (*Pkhd1*^*lsl/lsl*^) and kidneys from *Pkhd1*^*del67/del67*^ mice lacking the MLS, the renal tubules have significant mitochondrial structural defects by electron microscopy; (iii) in cell culture, reintroducing ICD_15_ partially restores the impaired mitochondrial function in CRISPR-mediated *Pkhd1* knockout in mIMCD3 cells; and (iv) digenic mice lacking both functional FPC and PC1 (*Pkd1*^*v/v*^) develop severe cystic disease in the kidney and pancreas *in utero*, a phenotype that closely resembles that observed in *Pkd1* null mice.

Taken together, these data establish a mitochondrial role for FPC and support a functional interaction between FPC-CTT and PC1-CTT within mitochondria that is important in maintaining the integrity of renal tubular architecture (Fig. 3).

### Cell adhesion

The cardinal feature of ARPKD is fusiform dilatation of the distal nephron and collecting ducts, suggesting a defect in tubular differentiation and the maintenance of tubular structural integrity. While the experimental studies reported to date have all shown adhesion abnormalities in renal tubular cells with FPC loss/knockdown, the experimental findings are conflicting [70, 71] and further studies are needed to elucidate the functional role of FPC in regulating cell adhesion and tubular integrity.

### Regulation of inflammation and fibrosis

Fibrosis is a histopathological hallmark of ARPKD-related renal cystic disease and CHF, the associated biliary lesion. Recent data implicate a direct link between FPC deficiency and immune-mediated fibrosis. Locatelli et al. [72] have shown that cholangiocytes derived from *Pkhd1*^*del4/del4*^ livers secrete chemokines, which in turn stimulate macrophage recruitment. These macrophages produce pro-inflammatory cytokines, which cause cholangiocytes to upregulate pro-fibrogenic signaling. In a second study using a human iPS-derived cholangiocyte 3D culture system, loss of FPC function promoted cholangiocyte proliferation and the production of connective tissue growth factor (CTGF) in an interleukin-8 (IL-8)-dependent manner [73]. These data suggest that FPC loss drives IL-8 and CTGF production, and these cytokine factors play a key role in CHF pathogenesis, highlighting the role of immune system dysregulation in ARPKD liver disease.

#### Translational relevance

These experimental data suggest that FPC and its protein domains play key roles in several cellular processes. Further defining the process-specific functions of FPC holds potential for identifying new druggable targets for therapeutic intervention.

## Conclusions and proposed FPC functional model

Taken together, the available data indicate that *PKHD1* is a phylogenetically new gene which encodes an evolutionally novel protein that is primarily expressed in terrestrial vertebrates. In his elegant description of vertebrate kidney functional evolution, Homer W. Smith observed “our kidneys constitute the major foundation of our physiological freedom” (*From Fish to Philosopher*, 1953). The fibrocystin-polyductin family, including FPC and its ancestral FPC-like (FPC-L) protein, may provide a prototypic example of protein evolution that occurred with vertebrate transition from aquatic to semi-terrestrial ecosystems and enabled vertebrate survival on land.

FPC is a complex protein that is targeted to the primary cilium and undergoes complex proteolytic processing to produce peptides that function in both extracellular and intracellular vesicles, as well as the cilia, nucleus, and mitochondria. While the specific functions of FPC remain incompletely understood, its functional roles appear to differ between rodents and humans, at least in renal epithelia. Yet the functional enigmas that have bedeviled investigators for the past two decades may be yielding to new insights.

Emerging evidence suggests that while defects in human *PKHD1* are necessary and sufficient to cause ARPKD kidney disease, in the mouse, *Pkhd1* defects are necessary, but disease expression also requires reduced *Pkd1* gene dosage or the loss of cystin. In other words, kidney disease in human ARPKD is a true monogenic disorder, while in the mouse, it is likely a digenic disorder requiring reductions in both functional FPC and PC1, and perhaps other cystoproteins. The key to this inter-species difference may lie in the limited sequence conservation of the FPC-CTT, which seems to have multiple cellular functions from ciliary sensing to proteome maintenance to mitochondrial functions and transcriptional regulation.

Building on the recent elegant work by Walker et al. [27], we propose a model for FPC function that incorporates many of the experimental findings summarized in this review (see Fig. 3).

In renal epithelia, FPC and PC1 are both localized to the primary cilium. Previous work has shown that PC1 represses the cilia-dependent cyst activation (CDCA) signal [74] and recent studies by Walker et al. extend this negative regulation to include FPC – specifically the FPC-CTT. In response to the binding of extracellular ligand(s), FPC undergoes Notch-like processing and additional enzymatic cleavage to release the FPC-CTT and produce the ICD_15_ product, which then translocates from the cilium to mitochondria. The FPC-ICD_15_ fragment and the PC1-CTT act synergistically to inhibit further the propagation of the CDCA, thereby maintaining the normal tubular diameter.

In the *cpk* mouse, the loss of cystin causes post-translational loss of FPC, which may contribute to renal cystogenesis by disrupting the FPC ciliary-mitochondrial pathway. Loss of cystin also releases negative regulation on necdin, allowing increased *Myc* expression. While experimental data demonstrate that both cystin and the FPC-CTT translocate to the nucleus, there is no evidence of physical interaction between these proteins, leaving open the possibility that they may cooperate in functional complexes to regulate the transcription of *Myc* and other genes implicated in cystogenesis.

Finally, the FPC-CTT has also been implicated in regulating ubiquitination pathways, based on both molecular assays and single nucleus RNA-seq analyses. However, mice lacking the CTT do not express a histopathological phenotype, suggesting that full expression of the ubiquitination defect may require additional, yet to be determined, defects.

In summary, recent experimental data from mice support a cooperative functional interaction between the FPC-CTT and PC1 to prevent the initiation and propagation of the CDCA cystogenic signal. This model raises the possibility that such a functional interaction is not required in human renal epithelia and thus, the loss of one or the other proteins is sufficient to initiate cystogenesis. In this context, the primary value of mouse *Pkhd1* models may be to further investigate the interaction of FPC and PC1 in common cellular processes and signaling pathways that are involved in tubular differentiation and maintaining tubular homeostasis.

## Key summary points

- ARPKD is primarily caused by defects in *PKHD1*-encoded FPC, a large multidomain single-pass transmembrane glycoprotein, which is highly expressed in the ductal epithelial of the kidney and the liver.
- FPC undergoes Notch-like processing. The cleavage fragments have extracellular and intracellular functions, the latter involving the primary cilium, the mitochondria, and the nucleus.
- Most patients with ARPKD carry two distinct pathogenic *PKHD1* alleles; unlike in *CTFR*, there are no mutational hot spots per se in *PKHD1*. Patients with two truncating *PKHD1* variants typically have a severe phenotype leading to perinatal or neonatal mortality. In contrast, in patients with one or two missense variants, the kidney and liver disease severity appears to be dictated by the *position* of the *PKHD1* variant.
- Defects in FPC are necessary and sufficient to cause human ARPKD. Whereas, in FPC-deficient mice, kidney disease expression also requires reduced dosage of other cystoproteins, e.g., PC1 or cystin. The primary value of mouse *Pkhd1* models may be to further elucidate the interaction of FPC and PC1 in common cellular processes that could help inform therapeutic development.
- Loss of FPC function results in reduced ubiquitination of downstream targets impacting cytoskeletal organization, TGF-β signaling, and retention of the epithelial sodium channel (ENaC) in the CD apical membrane, which may contribute to cystogenesis, fibrosis, and the hypertensive phenotype characteristic of ARPKD.

## Multiple choice questions

Answers appear following references

1. The N-terminus of FPC is more conserved evolutionarily than the C-terminus.
  True/False.
2. Which FPC domain harbors the ciliary targeting sequence (CTS), mitochondrial (MLS) and nuclear localization sequences (NLS)
  a. C-terminal tail (CTT)
  b. N-terminal domain
  c. TM domain
  d. IPT domain
3. FPC is proteolytically cleaved through a regulated, and Notch-like processing mechanism
  True/False.
4. Mature FPC has prominent roles in which of the following cell type in the kidney and the liver respectively
  a. mature, terminally differentiated ductal epithelia
  b. mesangial cells
  c. hepatocytes
  d. endothelial cells
5. While the function of FPC is incompletely understood, experimental data suggest that FPC plays key roles in (check all that apply):
  a. ciliary sensing and signaling
  b. transcriptional regulation and nuclear signaling pathways
  c. ubiquitination
  d. mitochondrial function
  e. cellular adhesion
  f. TGFb signaling pathway

## Supplementary Information

Below is the link to the electronic supplementary material.

ESM 1 (PPTX 723 KB)
