# Supplementary material for: Fibrocystin/polyductin (FPC): new functional insights into ARPKD pathogenesis revealed by informatics, comparative genomics, and model systems
Source: Pediatr Nephrol. 2026 Jan 28;41(9):2785–98. doi: 10.1007/s00467-025-07129-x (PMC13424714; doi:10.1007/s00467-025-07129-x)
Supplement: Supplementary file 1 — (PPTX 723 KB) [file 467_2025_7129_MOESM1_ESM.pptx]

## Slide 1
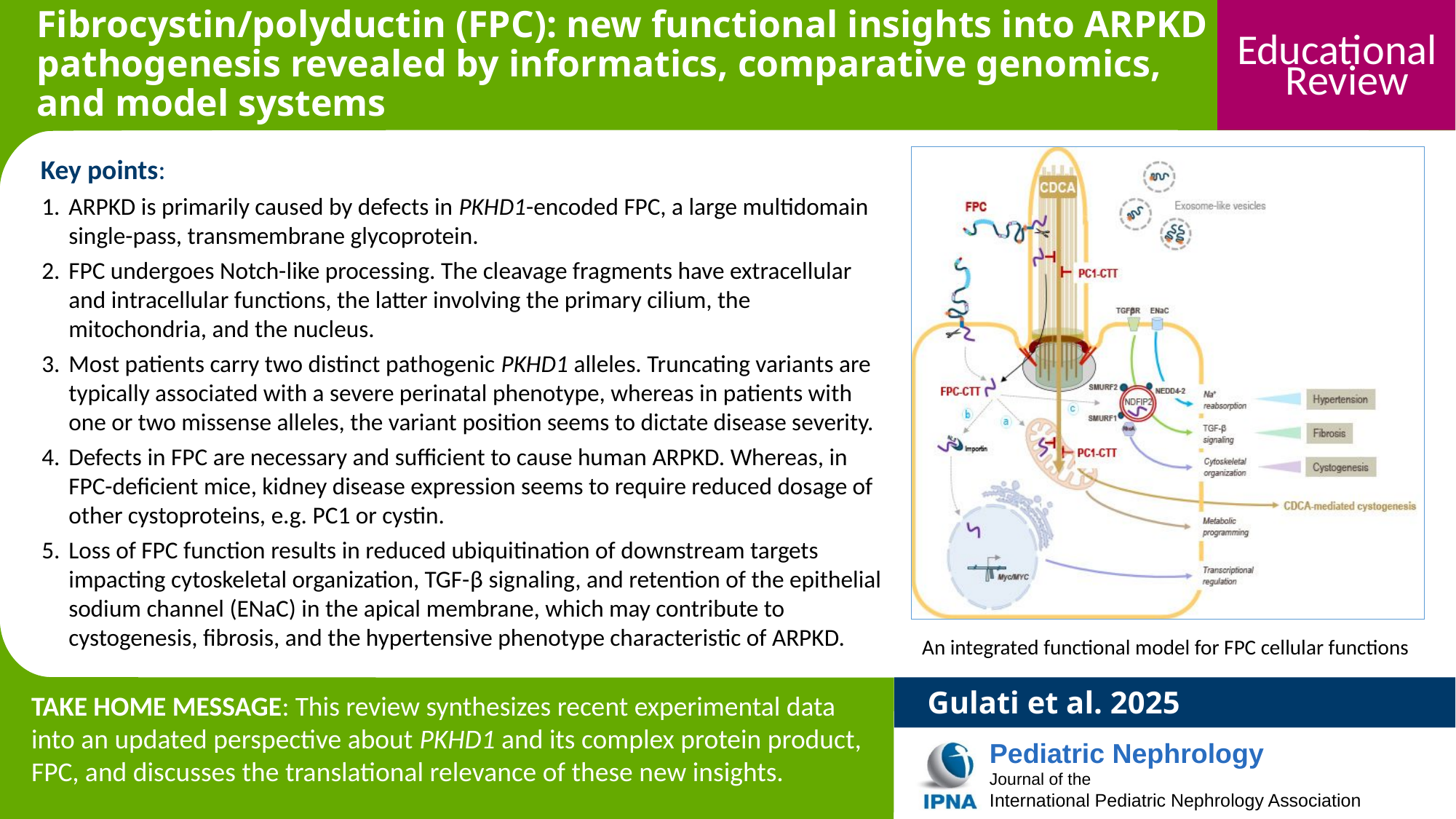

Fibrocystin/polyductin (FPC): new functional insights into ARPKD pathogenesis revealed by informatics, comparative genomics, and model systems
Key points:
ARPKD is primarily caused by defects in PKHD1-encoded FPC, a large multidomain single-pass, transmembrane glycoprotein.
FPC undergoes Notch-like processing. The cleavage fragments have extracellular and intracellular functions, the latter involving the primary cilium, the mitochondria, and the nucleus.
Most patients carry two distinct pathogenic PKHD1 alleles. Truncating variants are typically associated with a severe perinatal phenotype, whereas in patients with one or two missense alleles, the variant position seems to dictate disease severity.
Defects in FPC are necessary and sufficient to cause human ARPKD. Whereas, in FPC-deficient mice, kidney disease expression seems to require reduced dosage of other cystoproteins, e.g. PC1 or cystin.
Loss of FPC function results in reduced ubiquitination of downstream targets impacting cytoskeletal organization, TGF-β signaling, and retention of the epithelial sodium channel (ENaC) in the apical membrane, which may contribute to cystogenesis, fibrosis, and the hypertensive phenotype characteristic of ARPKD.
An integrated functional model for FPC cellular functions
Gulati et al. 2025
TAKE HOME MESSAGE: This review synthesizes recent experimental data into an updated perspective about PKHD1 and its complex protein product, FPC, and discusses the translational relevance of these new insights.
